# Noninvasive monitoring of plant-based formulations on skin barrier properties in infants with dry skin and risk for atopic dermatitis^☆^^☆☆^

**DOI:** 10.1016/j.ijwd.2017.10.009

**Published:** 2018-01-05

**Authors:** L. Lünnemann, L. Ludriksone, M. Schario, S. Sawatzky, A. Stroux, U. Blume-Peytavi, N. Garcia Bartels

**Affiliations:** a Dermatologic Practice Mahlow, Berlin, Germany; b Clinical Research Center for Hair and Skin Science, Department for Dermatology and Allergy, Charité-Universitätsmedizin Berlin, Berlin, Germany; c Department of Medical Statistics and Clinical Epidemiology, Charité-Universitätsmedizin Berlin, Berlin, Germany

**Keywords:** infants, dry skin, atopic dermatitis, skin barrier, emollient, skin potential of hydrogen

## Abstract

**Background:**

Dry skin and the associated impaired epidermal barrier function are postulated to constitute a major element in the development of atopic dermatitis.

**Objective:**

The aim of this study was to evaluate the effect of two plant-based formulations on the epidermal barrier function in a defined cohort of infants with a predisposition for atopic dermatitis.

**Methods:**

Over a period of 16 weeks, 25 infants who were ages 3 to 12 months and had an atopic predisposition and dry skin received two emollients that contained pressed juice of the ice plant. The infants received both cream and lotion on the forearm, only cream on the face, and only lotion on the leg. Stratum corneum hydration (SCH), transepidermal water loss (TEWL), skin surface pH, and sebum were assessed on the infants’ forehead, leg, and forearm. The Scoring Atopic Dermatitis (SCORAD) index was used for the clinical assessment.

**Results:**

SCH significantly increased in all body regions that were assessed. The forearm and leg revealed stable levels of pH and TEWL, but a decline in pH (week 16) and TEWL (week 4) was noted on the forehead. At week 16, sebum levels were lower on the forehead compared with those at baseline. SCORAD scores improved significantly during the study.

**Conclusion:**

A daily application of both emollients was associated with increased SCH levels and a stable course of TEWL, pH, and sebum on the forehead except for the forehead when compared with the forearm and leg. Clinically, improved SCORAD scores were noted.

## Introduction

Daily emollient therapy of the entire body from birth onward may represent a safe and efficacious strategy to prevent atopic dermatitis (AD; Simpson et al., 2010, Simpson et al., 2014). In nearly 60% of patients, AD manifests at the age of 3 to 6 months (Irvin and Miller, 2015), and dry skin is often the first morphologically recognizable symptom (Cork et al., 2009). Infants with at least one parent who has AD in particular are at a high risk (Williams et al., 2012). Appropriate emollient therapy during infancy is conjectured to be of particular relevance to delay the onset or even prevent AD (Blume-Peytavi et al., 2009, Cork et al., 2009, Proksch and Lachapelle, 2005, Simpson et al., 2010).

Dry skin is characterized by reduced water content in the stratum corneum (SC) that entails abnormal enzymic and mechanical properties (Rawlings and Matts, 2005). The two pivotal mechanisms that maintain an equilibrium state of SC hydration (SCH) include water retention by the hygroscopic components of the natural moisturizing factor complex and controlled transcutaneous water flux (Fluhr et al., 2012). Both have been shown to be impaired in dry skin conditions (Loden, 2003, Rawlings and Matts, 2005).

Moreover, an association between dry skin and a diminished barrier function has been previously suggested (Loden, 2003). The barrier impairment facilitates the penetration of exogenous irritants through the skin with a subsequent cutaneous inflammatory response (Rawlings and Matts, 2005, Tagami et al., 2006). Furthermore, both mechanisms reportedly undergo a dynamic adaption process throughout infancy, which causes lower natural moisturizing factor amounts and higher transepidermal water loss (TEWL) rates in the SC of infants compared with those of adults with normal skin (Fluhr et al., 2012, Nikolovski et al., 2008). Skin hydration increases during the first 30 days of life in neonates and is significantly higher in infants ages 3 to 12 months compared with adults (Garcia Bartels et al., 2009, Garcia Bartels et al., 2010, Garcia Bartels et al., 2011, Lavender et al., 2013)

Adequate skin care may enhance the SC barrier properties and thereby reduce the progression of AD or even prevent AD. Application of oils to the skin of healthy newborns may have different effects on the skin barrier compared with those of newborns with an atopic predisposition (Cooke et al., 2016).

Skin care is common in healthy neonates and infants (Blume-Peytavi et al., 2016, Gao and Simpson, 2014). A trend toward “free-of” cosmetics is widely observed, which means that consumers tend to prefer cosmetics that are free of certain ingredients (e.g., parabens, endocrine disrupters such as phthalates). Moreover, parents tend to prefer products with natural ingredients for their children (Gao and Simpson, 2014). The inclusion of botanical extracts in dermatological skin care products and their usage is increasingly popular especially in patients with AD (Reuter et al., 2010, Stallings and Lupo, 2009). Hence, the scientific evaluation of the effects of botanical extracts on infant skin barrier properties is of particular importance (Kuller, 2016).

Emollients that contain pressed juice of the ice plant, *Mesembryanthemum crystallinum*, have been shown to improve SC hydration in patients with AD (Schario et al., 2014). This plant has been reported to have antioxidant and antibacterial effects and influence the physiology of human keratinocytes (Schario et al., 2014). A previous study suggested that a daily application of moisturizer in neonates without skin disease but at a high risk of developing AD reduced the cumulative incidence of AD significantly (Cowdell et al., 2012). However, a scientific approach to considering the effects of emollients on skin barrier in this risk group as assessed by clinical and biophysical parameters is still lacking (Moncrieff et al., 2013).

The prevention of the manifestation of AD is the focus of the Barrier Enhancement for Eczema Prevention (BEEP) trial that is currently investigating the preventive effect of daily emollient application on infants at risk for AD (Chalmers et al., 2017, Simpson et al., 2012). Our results underline the goals of the BEEP study. We aimed to investigate the impact of a daily application of a plant-based moisturizing lotion and cream with ice plant pressed juice (IPPJ) on the skin barrier function in infants ages 3 to 12 months who are at an increased risk to develop AD and clinically dry skin.

## Methods

### Study design and participants

This single center, prospective, open-label trial lasted 16 weeks and was conducted between September 2011 and October 2012. Infants ages 3 to 12 months with clinically dry skin, an increased risk of developing AD, and an Erlangen Atopic Score ≥ 4 points were included in the study. Infants with at least one parent who was afflicted with previously classified atopy (e.g., atopic eczema and/or allergic rhinitis, allergic asthma) were considered at an increased risk of developing AD (Fischer, 1997, Gehring et al., 1991) and those with visible desquamation that was accompanied with a rough tactile sensation were evaluated as having dry skin. Furthermore, parents were required to agree for their child to participate in four swimming sessions. The study exclusion criteria consisted of an acute exacerbation of AD during the last 4 months (defined as the need to apply local or systemic immunosuppressive medication for longer than 3 consecutive days), congenital defects, diabetes, thyroidal diseases, and immunodeficiency and skin disorders of a contagious nature or that affect the investigated biophysical skin parameters.

Written informed consent for each infant was obtained from the infant’s legal guardians after the nature of the study had been fully explained and before the initiation of any study-related activity. After enrollment, baseline data were collected during follow-up visits at weeks 4 (W4), 12 (W12), and 16 (W16). Parents were instructed to visit the study site in addition to regularly scheduled visits if their infant showed any type of rash. The trial was approved by the local ethics committee and conducted in compliance with the Declaration of Helsinki.

### Intervention

All children received daily skin care with two emollients. Both interventional products contained IPPJ, and both the body care lotion and intensive cream were manufactured and labelled by Dr. Hauschka Med (WALA Heilmittel GmbH, Bad Boll, Germany; Greco and Lindequist, 2010). The ice plant body care lotion consisted of aqua, *Mesembryanthemum crystallinum* extract, alcohol, *Simmondsia chinensis* oil, *Persea gratissima* oil, *Prunus amygdalus dulcis* oil, *Manihot utilissima* starch, cera alba, lanolin, lysolecithin, *Mangifera indica* seed butter, *Butyrospermum parkii* butter, *Daucus carota* extract, sucrose stearate, sucrose distearate, *Chondrus crispus* extract, glyceryl stearate, hectorite, xanthan gum, stearic acid, *Amyris balsamifera* oil, *Rosmarinus officinalis* extract, and sodium stearoyl lactylate. Ingredients of the Intensive Ice Plant cream are aqua, *Mesembryanthemum crystallinum* extract, *Persea gratissima* oil, glycerin, *Mangifera indica* seed butter, alcohol, tricaprylin, *Prunus amygdalus dulcis* oil, *Simmondsia chinensis* oil, *Sesamum indicum* oil, lanolin, cetearyl alcohol, bentonite, *Butyrospermum parkii* butter, *Daucus carota* extract, *Rosmarinus officinalis* extract, *Amyris balsamifera* oil, lysolecithin, gleceryl oleate, and xanthan gum. The cream had a higher concentration of natural lipids than the lotion.

Parents were instructed on the dosage and application mode at the time of inclusion in the study (Table 1). The lotion and cream were applied only on the forearms. The forehead served as a control area for the cream formulation and the leg for the lotion. No other emollients were allowed except for sunscreen lotion; however, parents were instructed to use physical sunscreen. Parents were advised to retain their routine bathing procedures with the usual cleansing products.

**Table 1:** Baseline characteristics of participants

Characteristic	Participants (n = 26)
Infant sex	
Female, n (%)	14 (53.8)
Male, n (%)	12 (46.2)
Premature birth, n (%)	7 (26.9)
Age (month) MW ± SD (Median) [Range]	7.27 ± 2.7 (8.0) [3-12]
Age of gestation (SSW) MW ± SD (Median) [Range]	37.27 ± 3.4 (39.0) [29-42]
Body length at V0 (m) MW ± SD (Median) [Range]	0.70 ± 0.05 (0.71) [0.6-0.8]
Weight (kg) MW ± SD (Median) [Range]	7.8 ± 1.6 (7.6) [5.0-11.5]
Breast fed, n (%)	25 (96.1)
Atopic dermatitis infant n (%)	4 (15.4)
Last vaccination time 4 month before inclusion, n (%)	17 (65.4)
Medication, n (%)	17 (65.4)
Regular skin care performed prior to inclusion, n (%)	25 (96.2)

MW, XXX; SD, standard deviation; SSW, XXX.

### Outcome variables and clinical evaluations

The primary outcome variable was SCH on the forearm. The secondary outcome variables were TEWL, pH, sebum content, and Scoring Atopic Dermatitis (SCORAD) index scores. TEWL, SCH, pH, and sebum were assessed noninvasively with the Tewameter TM 300, Corneometer CM 825, Skin-pH-Meter PH 905, and Sebumeter SM 815 (Courage & Khazaka, Cologne, Germany) in accordance with standardized protocols (Garcia Bartels et al., 2010) under standardized ambient conditions including room temperature of 22°C to 26°C, relative humidity of 40% to 60%, and adaption period of at least 10 minutes with the investigational areas uncovered by clothing.

Skin functional parameters were assessed at each visit on three defined investigational areas, including the forehead, right mid-volar forearm, and right mid-lateral thigh. The forehead was selected to serve as a control area because it is less frequently afflicted by irritant dermatitis compared with the cheeks or chin. Sebum was evaluated on the forearm and leg. The assessed skin sites were free of eczematous involvement. The skin condition was evaluated at each visit using SCORAD scores (except for erythema, edema/population, oozing/crust, excoriation, and lichenification, clinical symptoms of dryness are shown by the dryness intensity assessment). No stressful physical activities, bathing, or skin care were allowed within 12 hours prior to the measurements. Adverse events (AEs) and serious adverse events were assessed from the time of study enrollment until exit from the study.

### Statistical methods

Descriptive statistics included absolute and relative frequencies for categorial measurements and mean, standard deviation (SD), median, and range for quantitative measurements. For between-visit comparisons or comparisons between different body regions with regard to skin functional parameters and SCORAD scores, the Wilcoxon test was used. P values < 0.05 (two-sided) were considered significant. No Bonferroni correction was performed. All statistical analyses were performed with the commercially available software SPSS 23 (IBM, Armonk, North Castle, NY; Zeger et al., 1988).

Sample size calculation, which was conducted with the commercially available software nQuery Version 6.0 (Statsols, Boston, MA), was based on mean differences in the development of the primary endpoint SCH between baseline and W16 with 26 infants. A power of 80% with a difference in means of 5 (SD = 8) could be shown on a two-sided significance level of α = 0.05.

## Results

A total of 26 infants were enrolled in the study and data of 25 infants were analyzed. Baseline characteristics are shown in Table 1. The parents of one infant decided to stop participation in the study due to an infection of the skin.

There were no adverse events related to the applied skin care during the intervention course of 16 weeks, and the study product application did not have to be discontinued or adjusted except for one dropout due to the worsening of the skin condition as a result of a systemic viral infection.

During the study, 19 AEs were recorded at W4, 20 at W12, and 19 at W16, which represents at least one AE in one patient. The observed AEs were age-typical conditions, such as common cold, fever, diarrhea, otitis media, teething problems, and minor trauma; three patients developed skin affections (i.e., erythema on the cheeks, diaper dermatitis in form of papules in the gluteal area, and follicular papules on the breast and back). Two patients required inpatient treatment for 3 days, including one patient who showed symptoms of dehydration due to gastroenteritis and the other who was diagnosed with a viral infection. In both cases, the application of the study products was continued as part of the skin care regimen, and the study was completed.

The forearm represented the interventional area for the cream and lotion and the control areas were the forehead (cream) and the leg (lotion; Table 2). Skin functional parameters showed distinct results and development depending on the investigated body region.

**Table 2:** Modified daily dosage and application mode of emollients on body surface

Emollient	Lotion	Cream	
Body area	Entire body surface (except face)	Face	Forearms
Dosage	per pass^a^	fingertip-units^b^	
	2	1	2

a 1 pass = 1 g lotion

b 1 fingertip unit = 0.5 g cream

**Fig. 1:**
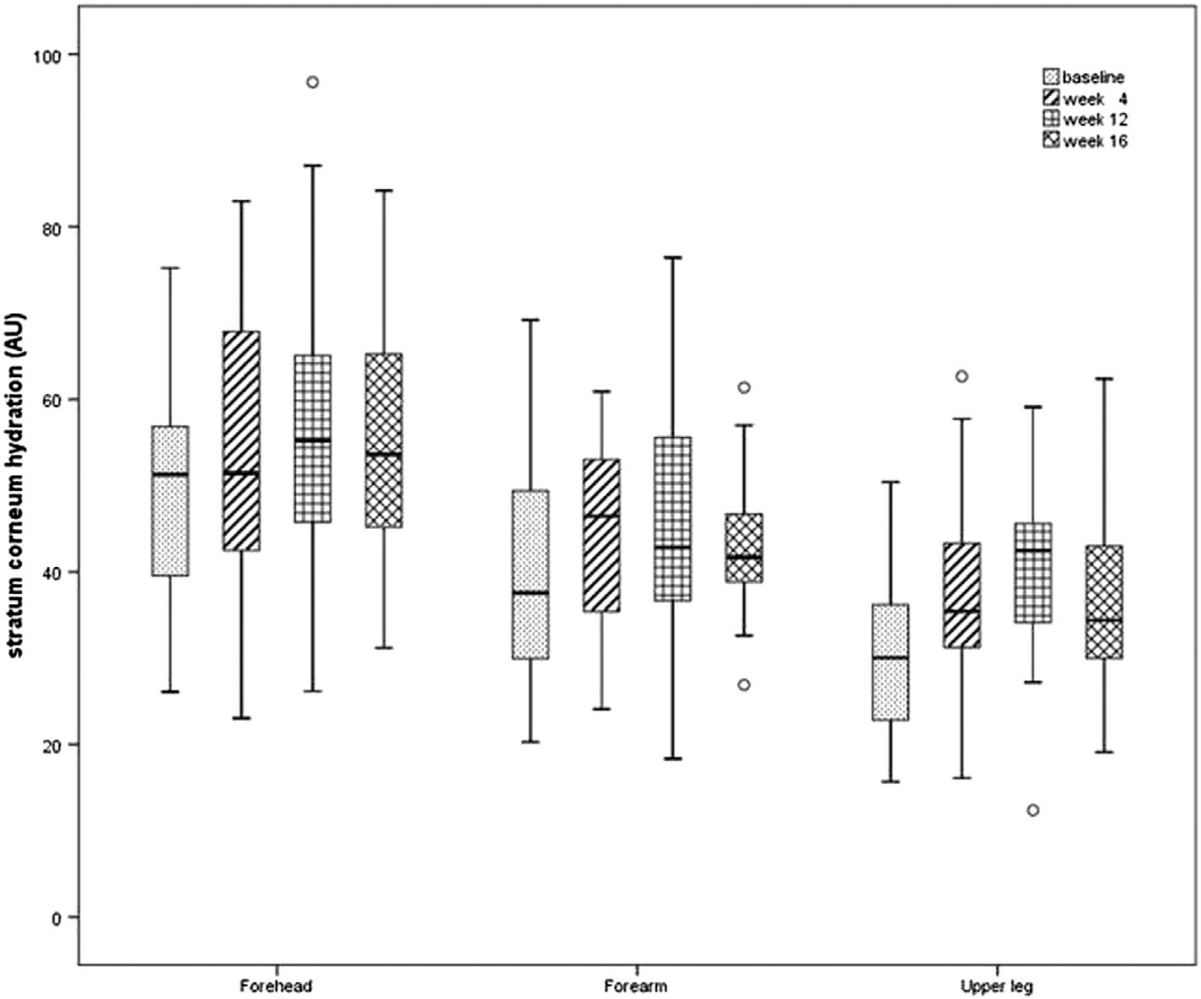
Stratum corneum hydration values on the forehead, forearm, and leg from baseline to week 16. In all three investigational areas, stratum corneum hydration levels were higher at the end of the study compared with those at baseline with increasing values until week 12. On the leg, a decline was noted at week 16. On the forearm, an increase in week 4 followed by a slight decrease in week 12 was observed. (o, outliers).

All investigated skin areas displayed higher SC hydration levels at the end of the study compared with the baseline levels. Until W12, a significant increase in SC hydration levels was observed on the forehead, forearm, and leg (*p* ≤ 0.026). On the leg, a subsequent decline (*p* = 0.032) at W16 was noted; however, the results remained higher (*p* = 0.042) compared with those from baseline. The forearm displayed an increase (*p* = 0.048) in SCH by W4 followed by a slight decrease in W12 at which time higher values were observed than those at baseline (Fig. 1).

**Fig. 2:**
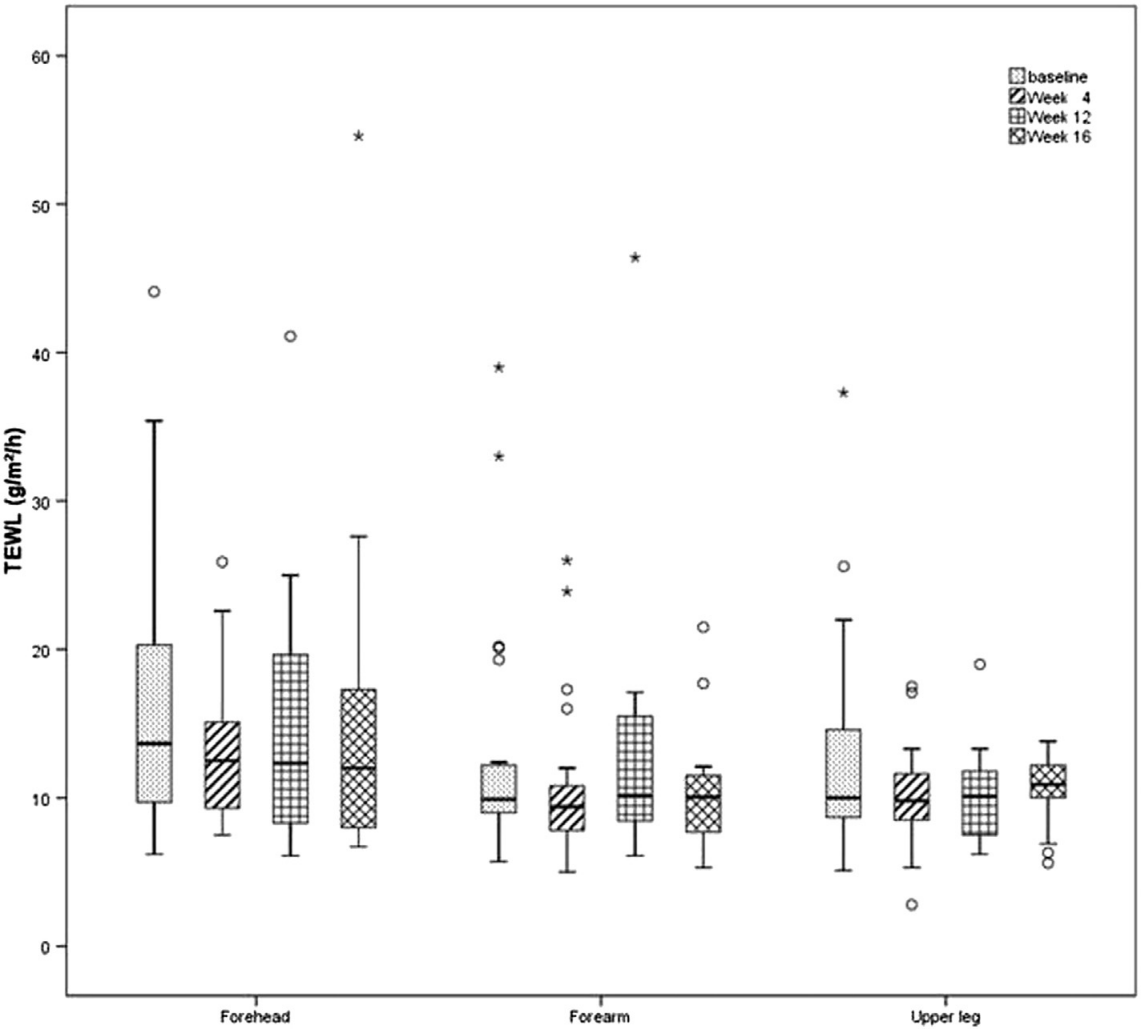
Transepidermal water loss values on the forehead, forearm, and leg from baseline to week 16. When comparing week 16 with baseline, the values remained stable in all investigational areas. On the forehead, decreasing values with a significant drop at week 4 were observed. (o/* outliers).

TEWL values remained stable at all measurement sites until W16 with comparable results at baseline. A gradual decline was observed on the forehead with a drop by W4 (*p* = 0.029). The forearm showed a trend toward decreased TEWL rates until W4 and then reached levels that were comparable to those at baseline by W12 (Fig. 2).

**Fig. 3:**
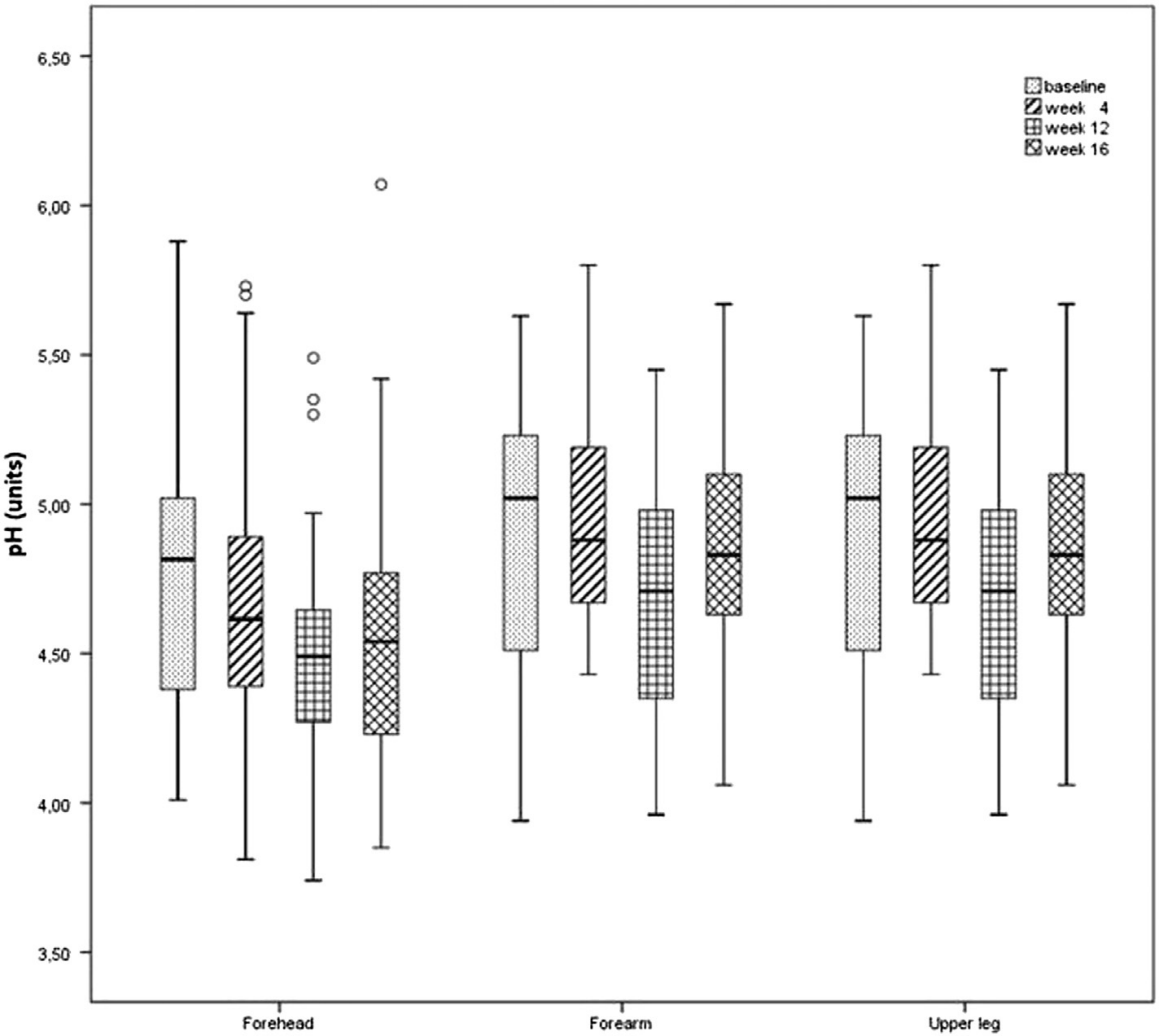
Skin surface potential of hydration on the forehead, forearm, and leg from baseline to week 16. Until week 12, a decline was observed on the forehead and forearm. When comparing week 4 and week 12, a significant drop was noted in all areas. On the forehead, values were significantly lower at week 16 compared with those at baseline. (o, outliers).

Skin surface pH values showed a decline on the forehead and forearm until W12. A significant drop was noted in all investigated areas when comparing W4 and W12 (p = 0.045 for forehead; p = 0.002 for forearm; p = 0.011 for upper leg). At W16, the pH values remained stable on the forearm and leg but displayed lower values on the forehead (*p* = 0.013) compared with those at baseline (Fig. 3).

At W16, sebum levels were significantly lower on the forehead compared with those from baseline (15.2 ± 19.6 vs. 5.0 ± 5.9; *p* = 0.003). The sebum course remained stable at a low level on the upper leg (1.3 ± 2.4; 0.8 ± 1.9; *p* > 0.326).

**Fig. 4:**
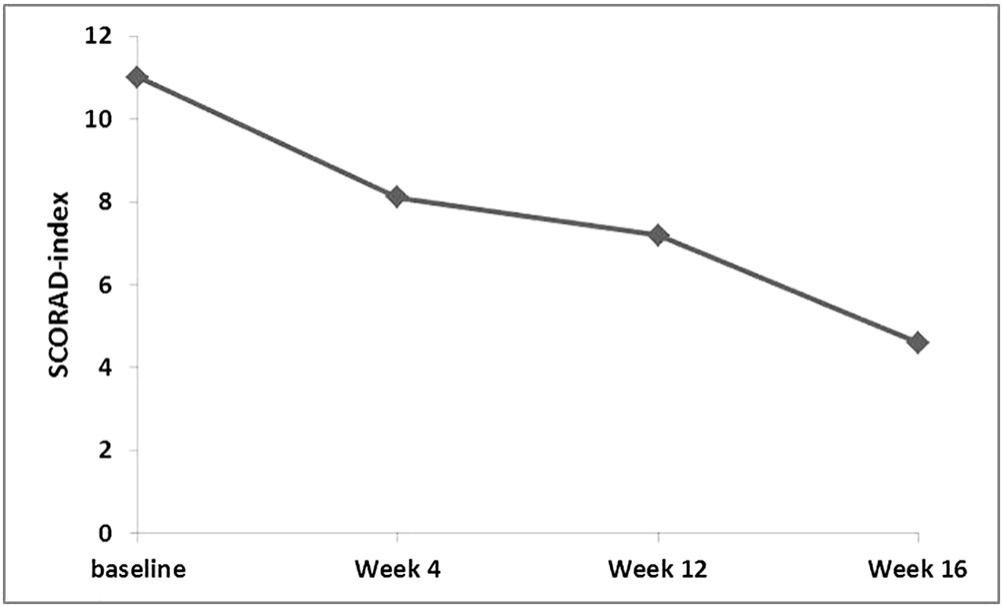
Development of Scoring Atopic Dermatitis index from baseline to week 16.

**Fig. 5:**
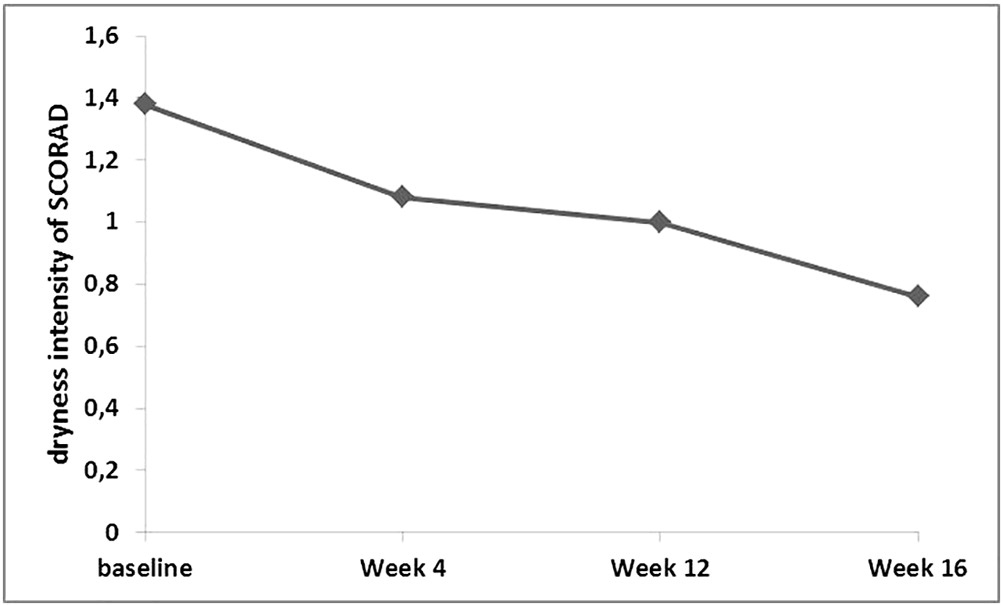
Dryness intensity assessment of Scoring Atopic Dermatitis index from baseline to week 16.

The SCORAD index (11.0 ± 6.8) and the dryness intensity assessment of the SCORAD (1.38 ± 0.57) declined significantly from baseline until W16 (4.6 ± 5.7; *p* ≤ 0.032; 0.76 ± 0.52; *p* ≤ 0.035), which reflects a reduction in erythema, dryness, and sleep loss (Fig. 4, Fig. 5).

## Discussion

The SC barrier is reportedly in a continuous maturation process beyond the neonatal period (Fluhr et al., 2013). A barrier that promotes the effect of an emollient application has been analyzed by previous studies in healthy neonates with normal skin (Garcia Bartels et al., 2010). Similar to the previous studies, a control group without topical treatment was not feasible in our study population due to the patients’ dry skin and high risk of developing AD (Williams et al., 2012). Moreover, a sufficient amount of data on untreated healthy infantile skin barrier parameters under comparable conditions is available (Gao and Simpson, 2014, Ludriksone et al., 2014, Sawatzky et al., 2016). Therefore, two formulations on different body areas were chosen to identify their effect on the skin barrier function in this specific dermatological risk group. Given the ongoing maturation process beyond the neonatal period and the reduced barrier properties, hydration- and SC-barrier-enhancing interventions are of particular significance in infants with dry skin and an atopic predisposition (Sawatzky et al., 2016, Schario et al., 2014).

The present study revealed an improvement trend of the initially lower SC hydration levels on the forearm and leg that received a daily application of cream and lotion or lotion, respectively. SCH rates on the forehead, which were higher than on the other areas before treatment, displayed a slight increase over time due to the daily application of the cream. Whether the moisturizing effect can be attributed to the two emollients and/or the hygroscopic properties of IPPJ compounds cannot be definitely concluded from these results.

Another aspect that is associated with SC water content is desquamatory enzyme activity (Rawlings and Matts, 2005). Corneodesmosome degradation in dry skin is reduced, which leads to retention and subsequent aggregation of corneocytes on the skin surface and results in scale formation (Rawlings and Matts, 2005). Consequently, SCH potentially reduces the dry clinical impression by normalizing desquamation. The observed superior SCH on the forehead might be related to higher sebum excretion compared with those from other investigated areas. Although the sebum levels in our cohort were relatively low as expected in accordance with the progressive decrease during the first months of life, the levels might be sufficient to influence the hydration state. However, data on the role of sebaceous lipids in SCH still remain controversial (Rawlings and Matts, 2005).

TEWL remained stable in all investigated areas throughout the study and displayed values that were comparable with those that were previously reported in infants without cutaneous diseases (Visscher et al., 2002). Thus, the inside-out epidermal permeability barrier in our cohort was indicated to be competent despite the presence of a xerotic skin condition. This observation is in contrast with recent data that reported increased TEWL values in infants with clinically dry skin even in the absence of eczema (Flohr et al., 2010). Hence, our results suggest that skin barrier alterations in infants with dry skin are not necessarily reflected by transepidermal water flux measurements. Consequently, TEWL assessments for skin barrier function monitoring in dry skin conditions in infants should be complemented with additional biophysical measurements.

The acid mantle is another crucial factor for the formation of a functionally competent SC barrier (Chan and Mauro, 2011). All three investigated areas displayed skin pH levels (Ali and Yosipovitch, 2013) that ranged between 4.5 and 5.2. A significant decrease during the investigational period was observed on the forehead but skin pH levels remained stable on the forearm and leg. Skin pH assessments in general are an important element to evaluate barrier integrity. Its significance lies in the associated cutaneous functions, most prominently the antimicrobial defense and enzymatic lipid processing for the formation of an effective permeability barrier (Ali and Yosipovitch, 2013, Chan and Mauro, 2011). Accordingly, AD displays a disrupted SC barrier function, which is characterized by a deviation from the characteristic acidic skin surface levels (Rippke et al., 2004, Schmid-Wendtner and Korting, 2006).

In contrasting, the measurement sites in our cohort of infants at a high risk of developing AD displayed normal skin acidity values. The improvement of the biophysical skin barrier parameters was accompanied with a clinical improvement of the skin condition as measured by SCORAD scores. Although no particular score for infants with dry skin and at risk for AD is available, the SCORAD index was used to reflect xerosis-related symptoms by quantifying the dryness intensity (Sawatzky et al., 2016, Schario et al., 2014).

The use of traditional plant-based skin care on infant skin is increasingly popular (Gao and Simpson, 2014, Panahi et al., 2012). The ice plant is traditionally used as a food product and consumed, for example, as a salad. Other fields of application include traditional medicine and skin care (Bouftira et al., 2008, Deters et al., 2012, Hanen et al., 2009, Lee et al., 2013, Wende et al., 2006). Ingredients from the fresh pressed juice of *Mesembryanthemum crystallinum* are related to natural moisturizing factors and considered to influence the cell physiology of human kerationcytes and fibroblasts (Deters et al., 2012). No sensitization or allergic reactions have been reported to date (Wende et al., 2006) but possible risks in long-term treatment with plant extracts cannot be precluded.

To further evaluate the effects of a controlled, environmental stress factor on skin conditions, infant swimming was performed once weekly (W4-W8). Previous data have shown an increased vulnerability of the SC water-holding capacity in patients with AD when their skin is exposed to chlorinated water (Seki et al., 2003). However, the skin barrier remained stable between W4 and W12 in all investigational areas, as shown by the assessed biophysical measurements. This confirms the previous data that suggest that skin barrier promote the effects of lotion that is applied after swimming (Garcia Bartels et al., 2011).

## Conclusions

Our results show a clinical and biophysical improvement of the skin conditions. A previous single-arm study with a similarly constituted cohort of infants at a high risk for AD already concluded that early emollient therapy has a preventive effect as the observed AD incidence of 15% was lower compared with that in the literature (Simpson et al., 2010). Therefore, we opted against a control group in this special risk cohort. However, our study comprised of a small cohort and thus, the results are not generalizable and should be investigated in larger trials. Still, previous data on the effects of plant- and petrolatum-based emollients on the SC barrier function properties in older infants at risk to develop AD reported a comparable benefit (Sawatzky et al., 2016, Schario et al., 2014). Even though the group size was limited, our study reveals additional insights into the development of AD in a cohort of infants at a high-risk for AD by combining objective quantitative (noninvasive assessment of skin barrier parameters) and clinical data (SCORAD scores; Simpson et al., 2014).
